# RNA sequencing analysis reveals *PgbHLH28* as the key regulator in response to methyl jasmonate-induced saponin accumulation in *Platycodon grandiflorus*

**DOI:** 10.1093/hr/uhae058

**Published:** 2024-02-28

**Authors:** Wuhua Zhang, Jinzhu Zhang, Yingdong Fan, Jie Dong, Peng Gao, Wanzheng Jiang, Tao Yang, Daidi Che

**Affiliations:** College of Horticulture and Landscape Architecture, Northeast Agricultural University, Harbin 150030, China; Key Laboratory of Cold Region Landscape Plants and Applications, Harbin 150030, China; College of Horticulture and Landscape Architecture, Northeast Agricultural University, Harbin 150030, China; Key Laboratory of Cold Region Landscape Plants and Applications, Harbin 150030, China; College of Horticulture and Landscape Architecture, Northeast Agricultural University, Harbin 150030, China; Key Laboratory of Cold Region Landscape Plants and Applications, Harbin 150030, China; College of Horticulture and Landscape Architecture, Northeast Agricultural University, Harbin 150030, China; Key Laboratory of Cold Region Landscape Plants and Applications, Harbin 150030, China; College of Horticulture and Landscape Architecture, Northeast Agricultural University, Harbin 150030, China; Key Laboratory of Cold Region Landscape Plants and Applications, Harbin 150030, China; College of Horticulture and Landscape Architecture, Northeast Agricultural University, Harbin 150030, China; Key Laboratory of Cold Region Landscape Plants and Applications, Harbin 150030, China; College of Horticulture and Landscape Architecture, Northeast Agricultural University, Harbin 150030, China; Key Laboratory of Cold Region Landscape Plants and Applications, Harbin 150030, China; College of Horticulture and Landscape Architecture, Northeast Agricultural University, Harbin 150030, China; Key Laboratory of Cold Region Landscape Plants and Applications, Harbin 150030, China

## Abstract

*Platycodon grandiflorus (Jacq.)* A. DC, known for its saponin content, can potentially prevent and treat cerebrovascular diseases and COVID-19. Triterpenoid saponin biosynthesis in plants is enhanced by methyl jasmonate (MeJA) application. However, the underlying molecular mechanisms of MeJA-induced saponin biosynthesis remain unknown in *P. grandiflorus*. In the current study, exogenous application of 100 μmol/l MeJA was identified to be optimal for promoting saponin accumulation. RNA sequencing analysis demonstrated the *PgbHLH28* gene as a key regulatory factor responding to MeJA during saponin accumulation. Overexpression of *PgbHLH28* in *P. grandiflorus* increased saponin content, while silencing of *PgbHLH28* significantly inhibited saponin synthesis, suggesting that *PgbHLH28* acts as a positive regulator of saponin biosynthesis. Yeast one-hybrid and dual luciferase assays demonstrated that *PgbHLH28* directly bound to the promoters of *PgHMGR2* and *PgDXS2* to activate gene expression. *PgHMGR2* and *PgDXS2* transformation promoted saponin accumulation, while silencing of these genes inhibited saponin biosynthesis. This study determined that MeJA promoted saponin accumulation in *P. grandiflorus* by inducing *PgbHLH28* gene expression and activating downstream genes (*PgHMGR2* and *PgDXS2*) involved in saponin biosynthesis. In conclusion, a complex regulatory network governing saponin biosynthesis following MeJA treatment was elucidated, offering a theoretical foundation for enhancing saponin content and biosynthesis efficacy in *P. grandiflorus.*

## 1. Introduction

*Platycodon grandiflorus* (Jacq.) A. DC. is a perennial herbaceous plant [1]. Its roots are rich in saponins, the major active components and a key indicator for breeding. Saponins possess pharmacological properties that are effective against chronic inflammatory diseases such as asthma and tuberculosis [2]. As a traditional herbal medicine in the pharmacopeias of China, Korea and Japan [3], the role of *P. grandiflorus* and other traditional Chinese herbs in the treatment of diseases, particularly COVID-19, has been gaining interest, leading to an expanding range of applications and a significant increase in demand [4, 5]. However, several challenges in *P. grandiflorus’s* cultivation process include the lack of superior varieties and cultivation measures. Therefore, a complete understanding of the transcriptional regulatory mechanisms of saponin biosynthesis, combined with corresponding cultivation measures, will provide effective solutions for developing cultivars with high-efficiency saponins.

The triterpenoid saponins in *P. grandiflorus* belong to the oleanane type, which are synthesized primarily in the mevalonic acid (MVA) pathway [6]. Farnesyl pyrophosphate (farnesyl-PP) is synthesized from isopentenyl diphosphate (IPP) and then it undergoes catalysis via *squalene synthase* (*SS*), *squalene epoxidase* (*SE*), *farnesyl-diphosphate synthase* (*FPPS*), *oxidosqualene cyclase* (*OSC*), and other major structural genes to form the saponin skeleton [6–8]. Finally, various saponin monomers are formed via hydroxylation, oxidation, and glycosylation catalyzed by *cytochrome p450 monooxygenases* (*CYP450*) and *glycosyltransferases* (*GTs*) [3, 9]. To date, understanding is primarily limited to the functional genes (i.e. *SS*, *SE*, and *DS*) in saponin biosynthesis. However, the regulatory networks controlling saponin biosynthesis are still unclear and require further elucidation [1, 10, 11].

Methyl jasmonate (MeJA) is a jasmonic acid derivative and is an important regulator of plant growth and secondary metabolite biosynthesis [12]. The exogenous application of MeJA can upregulate enzyme expression involved in terpenoid biosynthesis (i.e. *SS*, *SE*, *β-AS*, and *HMGR*) to promote the accumulation of terpenoid compounds [7]. Along with the direct regulation of terpenoid biosynthetic structural gene expression, MeJA also enhances saponin compound accumulation via induction of transcription factors (TFs), including bHLH, AP2/ERF, bZIP, and WRKY [13, 14], thereby regulating downstream structural genes. The bHLH TF plays a vital role in synthesizing monoterpenes, sesquiterpenes, and diterpenes in response to MeJA. In *Arabidopsis thaliana*, the *MYC2* TF binds to the promoters of *TPS11* and *TPS21* genes, facilitating sesquiterpene formation [15]. In diterpenoid biosynthesis, the bHLH TF *DPF* in rice binds to the *cis*-acting element (N-box) located in *CPS2* and *CYP99A2* promoter regions, consequently enhancing the synthesis of diterpenoid toxins [16]. Along with participating in monoterpene and diterpene biosynthesis, bHLH TFs also play important regulatory roles in triterpenoid and tetraterpenoid biosynthesis. In cucumber, bitter leaf (Bl) and bitter fruit (Bt) belong to the bHLH TF family that participates in synthesizing a triterpenoid compound, cucurbitacin C [17]. Furthermore, bHLH TFs regulating triterpenoid biosynthesis have also been identified in plants such as *Panax notoginseng* [18], *Panax ginseng* [19], and *Betula platyphylla* [20]. Overexpression of the bHLH TF *PIF5* in *A. thaliana* T87 suspension culture cells increased the content of tetraterpenoid compounds, such as carotenoids [21].

Exogenous MeJA application can increase the saponin content in the roots of *P. grandiflorus*. However, since the mechanisms of transcriptional regulation are unknown and require further elucidation, a regulatory network of MeJA-induced saponin biosynthesis was characterized using transcriptome data. The *PgbHLH28* gene was identified as a key regulatory factor in MeJA-induced saponin biosynthesis. It binds to the promoter regions of saponin biosynthetic genes (*PgDXS2* and *PgHMGR2*) activating their expression levels and promoting saponin accumulation. These findings are of great significance in understanding the role of MeJA in regulating saponin accumulation via transcriptional regulation. This new understanding provides guidelines for increasing saponin content, enhancing biosynthesis efficacy, and facilitating molecular breeding of *P. grandiflorus* containing high saponin.

## 3. Results

### 3.1. Optimal MeJA concentration for saponin content induction in *P. grandiflorus*

**Figure 1:**
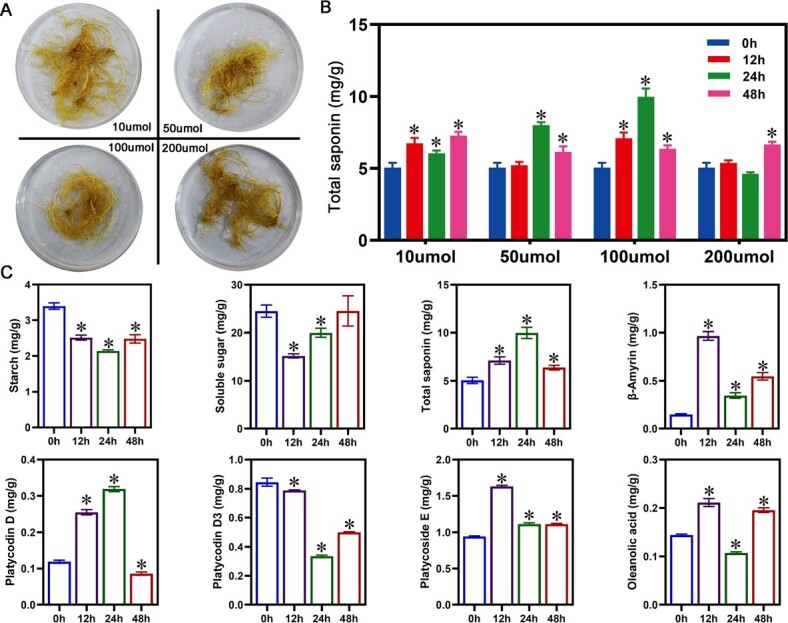
Changes in saponin content, saponin monomers, and intermediate synthesized products at different concentrations of MeJA. (A) Morphology of *P. grandiflorus* roots at four MeJA concentrations at 72 h. (B) Total saponin content at four MeJA concentrations. (C) Soluble sugars, starch, three saponin monomers, and intermediate products (β-amyrin and oleanolic acid) in *P. grandiflorus* roots treated with 100 μmol/l of MeJA.

MeJA functions as an inducer and signaling molecule in saponin biosynthesis [22]. In order to investigate the effect of exogenously applied MeJA on saponin accumulation, we designed four different concentrations of MeJA (10 μmol/l, 50 μmol/l, 100 μmol/l, and 200 μmol/l) to treat the roots of *P. grandiflorus*. After MeJA treatment, the degree of root browning increased with the concentration of MeJA (Fig. 1A). Saponin accumulation after treatments with four different concentrations MeJA was compared. The results demonstrated that MeJA could promote saponin accumulation, and 100 μmol/l of MeJA was the optimal concentration for promoting saponin accumulation (Fig. 1B). Therefore, 100 μmol/l of MeJA was selected as the optimal treatment concentration for further experiments. Soluble sugars and starch, acting as substrates in the glycolysis, directly impact the biosynthesis and accumulation of saponins via pathways such as MVA and 2-C-methyl-D-erythritol-4-phosphate (MEP). Under the optimal MeJA concentration treatment, soluble sugar and starch content decreased compared to the control (Fig. 1C). Furthermore, the levels of intermediate products (i.e. β-amyrin and oleanolic acid) in saponin biosynthesis were significantly enhanced (Fig. 1C). In addition to the upstream substrates and intermediate products, the two saponin monomers (platycodin D and platycoside E) were also induced by MeJA (Fig. 1C). These results demonstrated that exogenous application of MeJA could promote saponin accumulation, and 100 μmol/l of MeJA was the optimal concentration for promoting saponin accumulation.

**Figure 2:**
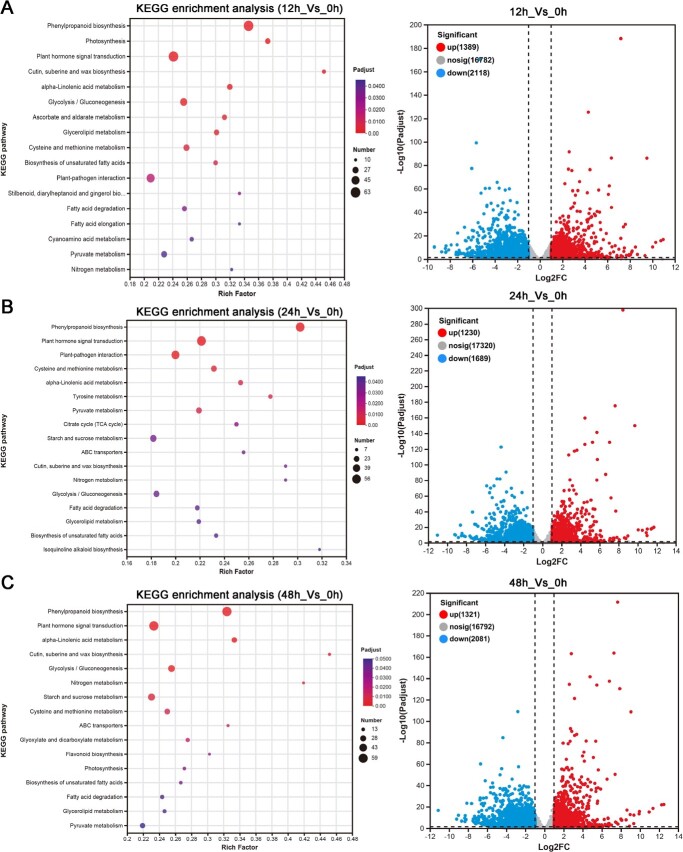
Volcanograms and KEGG enrichment analysis of DEGs in *P. grandiflorus* roots under 100 μmol/l MeJA treatment. (A) 12h_Vs_0h. (B) 24h_Vs_0h. (C) 48h_Vs_0h.

### 3.2. DEG and enrichment analyses

To investigate the molecular mechanism of MeJA-induced saponin biosynthesis in *P. grandiflorus*, transcriptome sequencing was performed on *P. grandiflorus* root samples treated with 100 μM MeJA at 0, 12, 24, and 48 h. A total of 96.37 Gb clean data were obtained. Alignment of the clean data with the *P. grandiflorus* genome produced alignment rates ranging from 94.34%–94.91% (Supplementary Table S1). DEG analysis identified a total of 5251 DEGs (1389 up and 2118 downregulated) in the 12h_Vs_0h comparison, 3133 DEGs (1230 up and 1689 downregulated) in the 24h_Vs_0h comparison, and 3401 DEGs (1321 up and 2081 downregulated) in the 48h_Vs_0h comparison. According to the KEGG enrichment results, the alpha-linolenic acid metabolism involved in MeJA biosynthesis, as well as the glycolysis/gluconeogenesis, starch and sucrose metabolism, and ABC transporters pathways involved in saponin biosynthesis and transport, were significantly enriched after MeJA treatment. Furthermore, plant hormone signal transduction pathways also showed significant enrichment (Fig. 2A–C). These results demonstrated that MeJA, as a hormone-signaling molecule, was found to induce triterpenoid saponin-related pathway gene expression, thereby promoting the accumulation of saponins in the roots of *P. grandiflorus*.

### 3.3. Genetic basis of *P. grandiflorus* saponin biosynthesis under MeJA treatment

**Figure 3:**
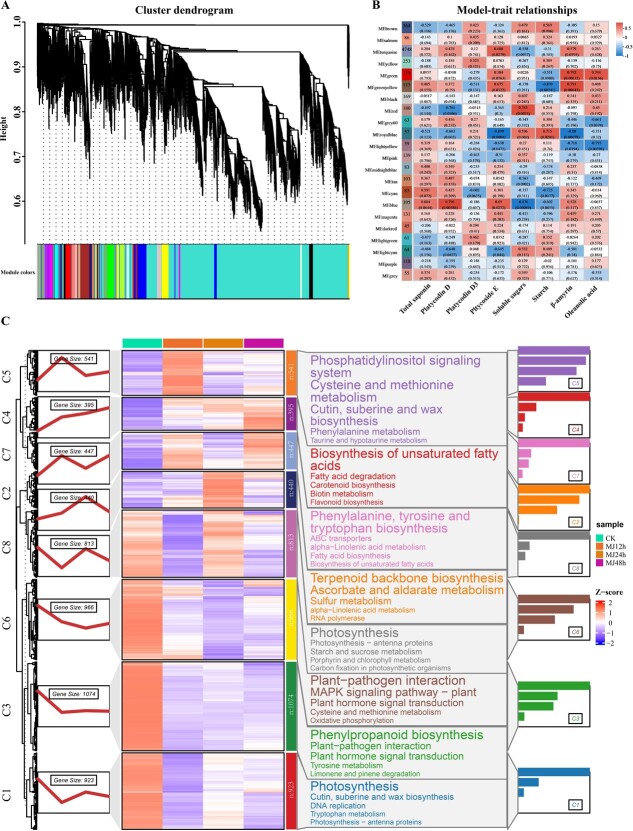
Transcriptome and metabolite correlation analysis of *P. grandiflorus* under MeJA treatment. (A) Dendrogram of the co-expression modules during saponin accumulation under MeJA treatment. The tree branches contain 22 modules, labeled with various colors. (B) Heat map displaying saponin correlation module. Different colors of each column denote specific modules. Each row corresponds to a metabolite. The positive relationship between the model and the content of various metabolites is indicated in red, and the negative correlation is in blue. (C) Temporal expression and KEGG enrichment of eight gene clusters.

Investigating the genetic foundation of saponin biosynthesis in *P. grandiflorus* under MeJA treatment yielded 7590 DEGs (average TPM > 1, CV > 0.1). In order to elucidate the regulatory mechanisms of saponin biosynthesis under MeJA treatment, the DEG co-expression networks were examined using the WGCNA package. Based on the expression patterns, all the DEGs were divided into 22 modules (Fig. 3A; Supplementary Table S2). The correlation analysis between the 22 modules and metabolites demonstrated a strong correlation between MEblue, MEturquoise, MEred, MEroyalblue, and MEgreen modules and β-amyrin, oleanolic acid, starch, and three individual saponin moieties. These results showed that the genes in these five modules were primarily involved in MeJA-induced saponin biosynthesis (Fig. 3B).

To further understand the MeJA response and saponin biosynthesis regulation of the five modules, these genes were subdivided into eight clusters using Mfuzz based on their expression pattern similarities (Fig. 3C; Supplementary Table S3). KEGG enrichment analysis of the eight clusters demonstrated that Cluster 2 exhibited significant enrichment in alpha-linolenic acid metabolism, the key biosynthetic pathway for MeJA biosynthesis. Cluster 2 was also enriched in the terpenoid backbone biosynthesis pathway, the major pathway for synthesizing of triterpenoid saponins, monoterpenes, and sesquiterpenes. These results demonstrated that the 440 genes within Cluster 2 were the core genes involved in MeJA-induced saponin biosynthesis.

### 3.4. Generation of saponin biosynthesis regulatory networks under MeJA treatment

**Figure 4:**
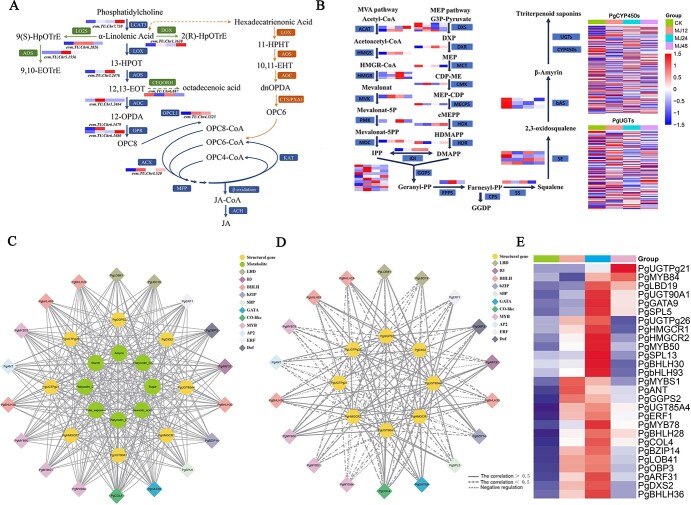
The regulatory network of saponin in *P. grandiflorus*. (A) Metabolic pathway for MeJA. (B) Metabolic pathway for saponin. (C) Green circles denote metabolites (including soluble sugar, starch, total saponin, β-amyrin, platycodin D, platycodin D3, platycoside E, and oleanolic acid). Yellow circles denote structure genes. Diamonds, each with a distinct color, represent various families of TFs identified in Cluster 2. (D) The regulatory network between structural genes and TFs of saponin biosynthesis under MeJA. (E) Heatmap of TF and structural gene expression involved in saponin biosynthesis in Cluster 2.

By analyzing the genetic basis of *P. grandiflorus* saponin biosynthesis under MeJA treatment, Cluster 2 was considered the main module in response to MeJA regulation of saponin synthesis. KEGG pathway analysis showed that Cluster 2 included alpha-linolenic acid metabolism and terpenoid backbone biosynthesis pathways, which are crucial for MeJA and terpenoid biosynthesis, respectively. Expression analysis of structural genes within alpha-linolenic acid and terpenoid backbone biosynthesis pathways showed MeJA could upregulate these genes (Fig. 4A–B; Supplementary Table S4). In order to generate a regulatory network for MeJA-induced saponin biosynthesis, eight structural genes (including *PgGGPS2*, *PgUGTPg21*, *PgUGT85A4*, *PgUGT90A1*, *PgUGTPg26*, *PgHMGCR1*, *PgHMGR2*, and *PgDXS2*) involved in saponin biosynthesis were identified, exhibiting highly correlated relationship with saponin metabolite content in Cluster 2 (Fig. 4C; Supplementary Table S5). At the same time, a total of 19 TFs (including bHLH, AP2/ERF, and Dof) were identified, which may promote saponin accumulation via regulating structural genes involved in saponin biosynthesis (Fig. 4D; Supplementary Table S5). The regulatory network of saponin accumulation induced by MeJA was generated by calculating the transcription accumulation pattern of TFs and structural genes related to saponin biosynthesis, as well as the potential binding affinity of TFs to structural gene promoters (Fig. 4D; Supplementary Table S5–S6). Network analysis identified that *PgbHLH28* was highly correlated with the structural genes involved in saponin biosynthesis (i.e. *PgGGPS2*, *PgHMGR2*, *PgHMGR1*, and *PgDXS2*), which was identified as the most promising candidate for further investigation.

### 3.5. *PgbHLH28* regulates saponin accumulation in *P. grandiflorus*

**Figure 5:**
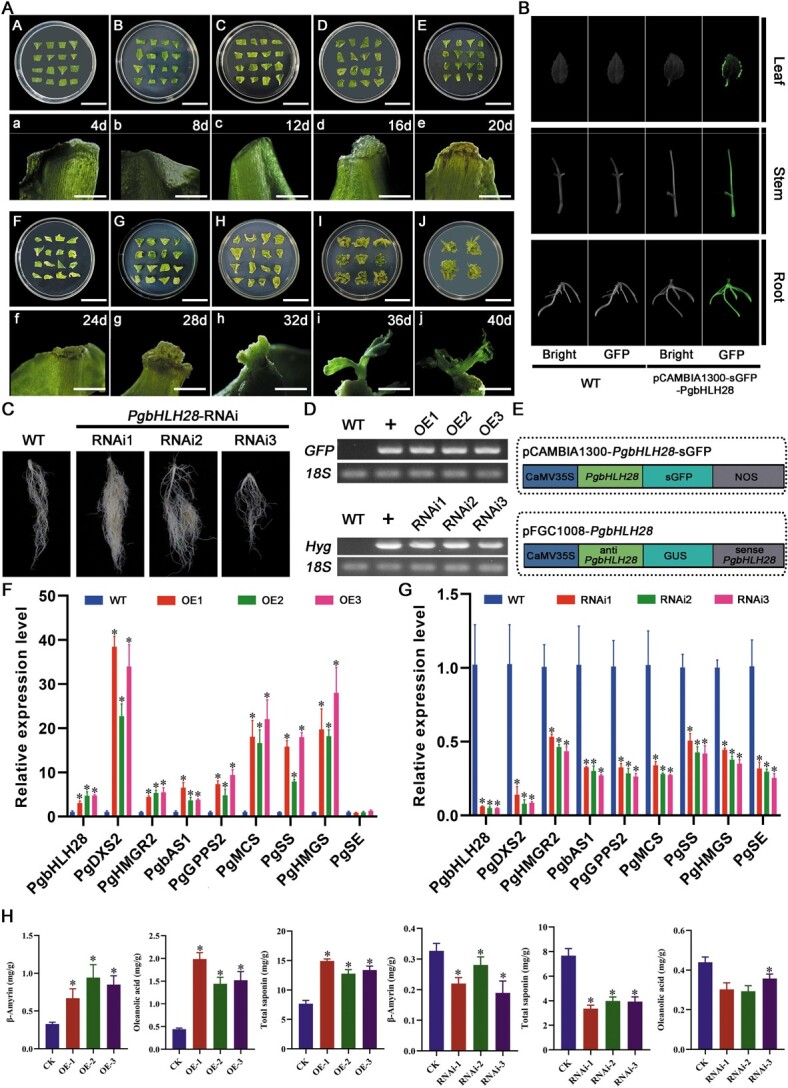
*PgbHLH28* modulates saponin accumulation by regulating genes involved in the saponin biosynthesis pathway. (A) Adventitious bud induction in *P. grandiflorus*. (B) GFP fluorescence detection in *PgbHLH28* overexpression lines. Scale bar: 3 cm (A–J), 5 mm (a–j) (C) Hairy root development via RNA interference of *PgbHLH28* in *P. grandiflorus*. (D) RT-PCR confirmation of transgenic plants. (E) Schematic diagram of the construction of overexpression and RNA interference vectors. (F) Relative expression levels of genes involved in saponin biosynthesis pathway in *PgbHLH28* overexpression lines. *PgbHLH28*-OE1, *PgbHLH28*-OE2, and *PgbHLH28*-OE3 are three independent *PgbHLH28* overexpression lines. (G) Relative expression levels of genes involved in saponin biosynthesis pathway in *PgbHLH28* silencing lines. *PgbHLH28*-RNAi1, *PgbHLH28*-RNAi2, and *PgbHLH28*-RNAi3 are three independent *PgbHLH28* silencing lines. (H) Total saponin, oleanolic acid, and β-amyrin content in overexpression and RNA silencing lines.

Through regulatory network analysis, the *PgbHLH28* gene was identified as the key regulator in response to MeJA-induced saponin accumulation in *P. grandiflorus*. In order to verify the function of the *PgbHLH28* gene, both overexpression and RNA interference vectors of the *PgbHLH28* gene were constructed to demonstrate its function in *P. grandiflorus* (Fig. 5E). Transgenic overexpression plants (Fig. 5A–B, D), overexpression transgenic hairy roots (Supplemental Material Fig. S1A–B), and RNA-silencing transgenic hairy roots (Fig. 5C–D) were generated via *Agrobacterium*-mediated transformation. β-amyrin, oleanolic acid, and total saponin levels were significantly increased in *PgbHLH28-*overexpressing plants and decreased in silenced lines compared to the wild-type (WT) plants (Fig. 5H; Supplemental Material Fig. S1C). The relative expression of *PgbHLH28* and other structural genes, such as *PgDXS2* and *PgHMGR2*, in the network involved in saponin synthesis was significantly upregulated in the *PgbHLH28-*overexpressing plants and downregulated in RNA-silencing transgenic hairy roots compared to WT (Fig. 5F–G; Supplemental Material Fig. S1D).

Measurements of saponin content and expression of structural genes involved in saponin biosynthesis in the *PgbHLH28* transgenic hairy roots and transgenic plants demonstrated that the results obtained from both methods were consistent (Fig. 5F–H; Supplemental Material Fig. S1C–D), suggesting that both methods could be used to validate *P. grandiflorus* gene functions. Furthermore, the hairy root transformation method offered transformation time and operability advantages. Therefore, the hairy root transformation method was selected as the primary approach to validate gene function in further experiments. These experiments established *PgbHLH28* as a positive regulator of saponin biosynthesis in *P. grandiflorus*.

### 3.6. *PgbHLH28* activates the transcription of downstream genes (*PgHMGR2* and *PgDXS2*)

**Figure 6:**
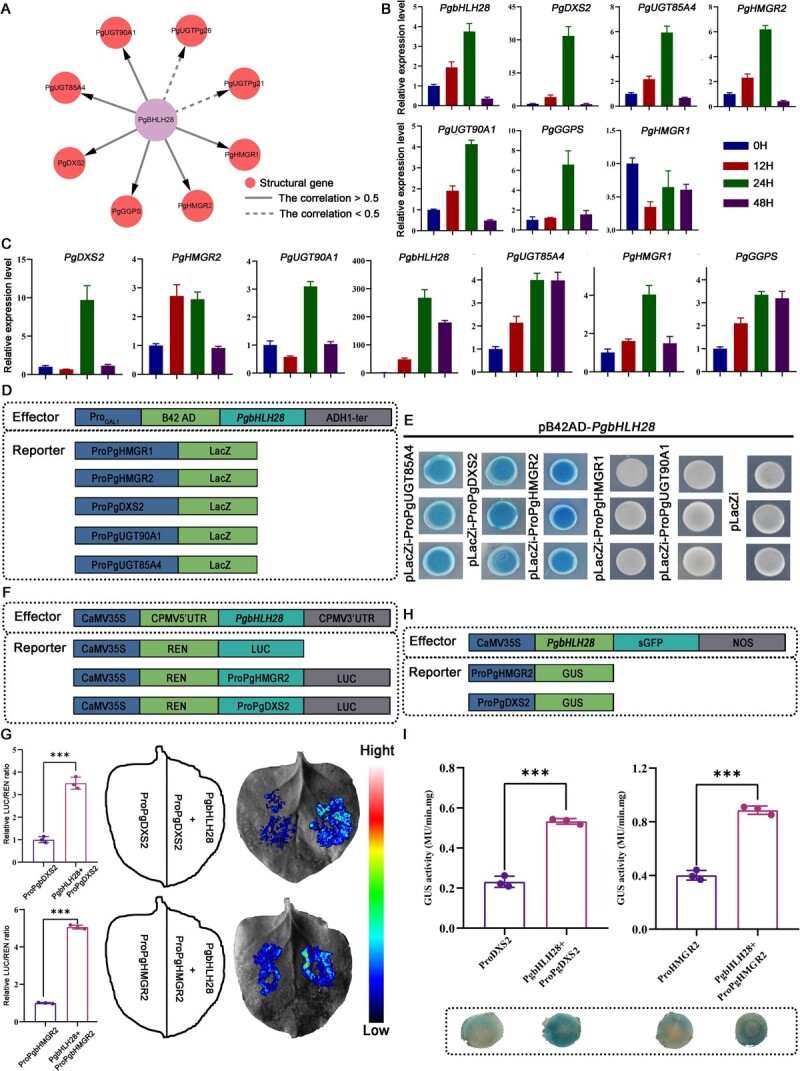
The *PgbHLH28* gene promotes the expression of the terpenoid backbone biosynthetic genes *PgDXS2* and *PgHMGR2*. (A) *PgbHLH28* is a key regulatory factor in the synthesis of terpenoid compounds. The pink circles denote structural genes. Solid lines represent a correlation >0.5 between *PgbHLH28* and the structural genes, while dashed lines represent a correlation <0.5. (B) RT-qPCR of *PgbHLH28* TF and six highly correlated structural genes under MeJA treatment. (C) Transient overexpression of *PgbHLH28* TF in *P. grandiflorus* roots increases the expression levels of six structural genes. (D) Schematic diagram of Y1H construction structure. (E) *PgbHLH28* binds to the promoters of *PgUGT85A4*, *PgDXS2*, and *PgHMGR2* genes. (F) Schematic diagram of dual-luciferase vector structure. (G) Dual-luciferase assays show *PgbHLH28* activating the transcriptional activity of *PgDXS2* and *PgHMGR2* genes. (H) Schematic diagram of GUS activity vector structure. (I) GUS reporter assays demonstrate *PgbHLH28* activating the transcription activity of *PgDXS2* and *PgHMGR2* genes.

Transgenic experiments identified *PgbHLH28* as a key regulator of MeJA-induced saponin accumulation in *P. grandiflorus*. Regulatory network analysis revealed structural genes (*PgGGPS2*, *PgHMGR2*, *PgHMGR1*, and *PgDXS2*) as downstream targets regulated by *PgbHLH28* (Fig. 6A).

In order to determine if the *PgbHLH28* gene regulates the structural gene, the pCAMBIA1300-*PgbHLH28*-sGFP overexpression vector was constructed and transiently overexpressed in the roots. The relative expression levels of *PgbHLH28* and six structural genes in the regulatory network were significantly upregulated, and MeJA could also induce the expression of these genes, as validated by qRT-PCR (Fig. 6B–C). Secondly, Y1H assays confirmed *PgbHLH28* binding to the *cis*-elements in the promoter regions of *PgDXS2*, *PgHMGR2*, and *PgUGT85A4* (Fig. 6 D–E). While PgHMGR2 and PgDXS2 are key rate-limiting enzymes involved in saponin biosynthesis, further investigations focused on the regulation between *PgbHLH28* and two structural genes (*PgHMGR2* and *PgDXS2*). Furthermore, dual-luciferase reporter assays in tobacco demonstrated that *PgbHLH28* significantly enhanced the transcriptional activity of *PgDXS2* and *PgHMGR2* compared to the control (Fig. 6F–G). The GUS staining experiment also demonstrated that *PgbHLH28* enhanced the transcriptional activity of *PgDXS2* and *PgHMGR2* genes (Fig. 6H–I). Taken together, these findings demonstrated that the *PgbHLH28* TF regulated the expression of the saponin biosynthesis pathway functional genes, *PgDXS2* and *PgHMGR2*, via directly binding to their *cis*-regulatory elements in the promoter regions, thereby modulating saponin accumulation.

### 3.7. *PgDXS2* and *PgHMGR2* regulate saponin accumulation in *P. grandiflorus*

**Figure 7:**
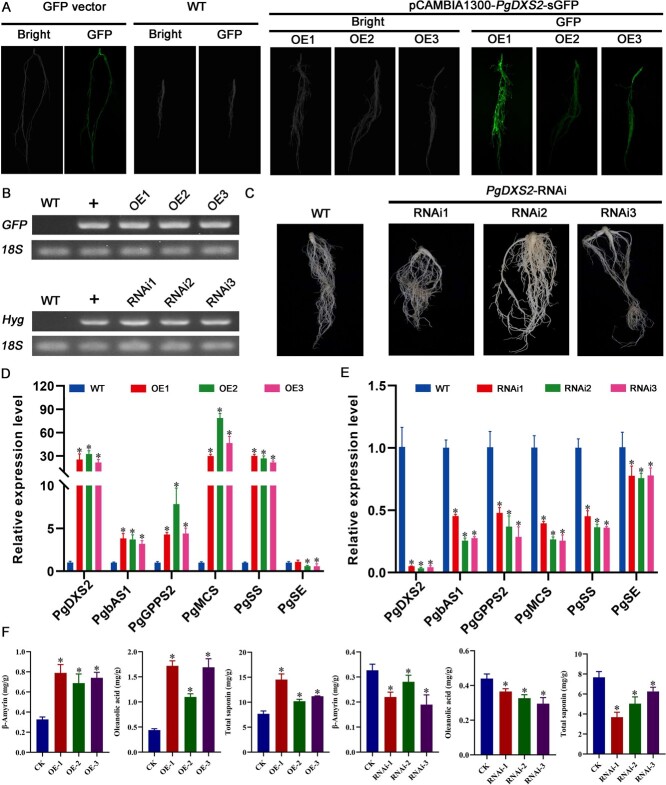
*PgDXS2* modulates saponin accumulation in *P. grandiflorus.* (A) Hairy root generation through overexpression of *PgDXS2* in *P. grandiflorus*. (B) RT-PCR validation of transgenic hairy root. (C) Hairy root generation via RNA interference of *PgDXS2* in *P. grandiflorus*. (D) Relative expression of genes involved in saponin biosynthesis in *PgDXS2* overexpression lines. *PgDXS2*-OE1, *PgDXS2*-OE2, and *PgDXS2*-OE3 are three independent *PgDXS2* overexpression lines. (E) Relative expression of genes involved in saponin biosynthesis in *PgDXS2* silencing lines. *PgDXS2*-RNAi1, *PgDXS2*-RNAi2, and *PgDXS2*-RNAi3 are three independent *PgDXS2* silencing lines. (F) Total saponin, oleanolic acid, and β-amyrin content in overexpression and RNA silencing lines.

**Figure 8:**
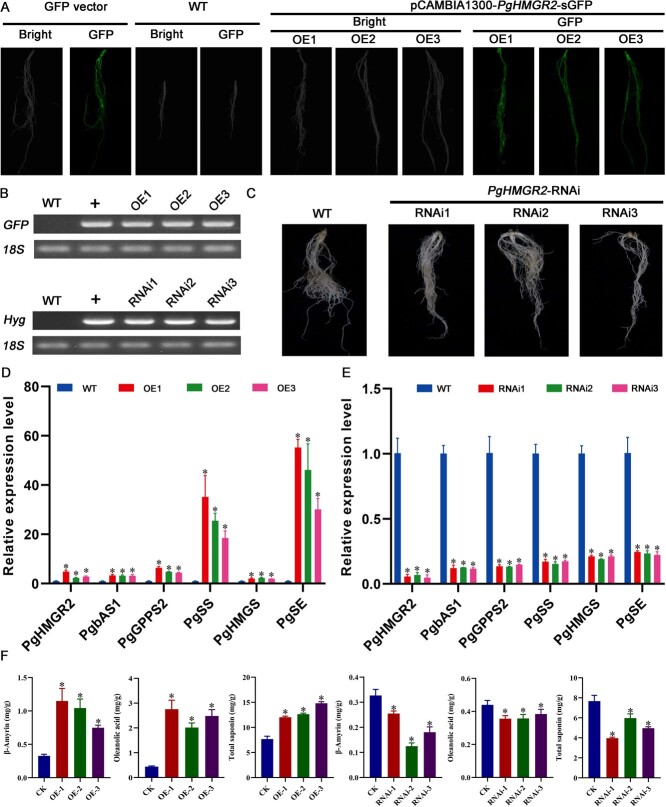
*PgHMGR2-*modulated saponin accumulation in *P. grandiflorus.* (A) Hairy root generation through overexpression of *PgHMGR2* in *P. grandiflorus*. (B) RT-PCR validation of transgenic hairy root. (C) Hairy root generation *via* RNA interference of *PgHMGR2* in *P. grandiflorus*. (D) Relative expression of genes involved in saponin biosynthesis in *PgHMGR2* overexpression lines. *PgHMGR2*-OE1, *PgHMGR2*-OE2, and *PgHMGR2*-OE3 are three independent *PgbHLH28* overexpression lines. (E) Relative expression of genes involved in saponin biosynthesis pathway in *PgHMGR2* silencing lines. *PgHMGR2*-RNAi1, *PgHMGR2*-RNAi2, and *PgHMGR2*-RNAi3 are three independent *PgHMGR2* silencing lines. (F) Total saponin, oleanolic acid, and β-amyrin content in overexpression and RNA silencing lines.

**Figure 9:**
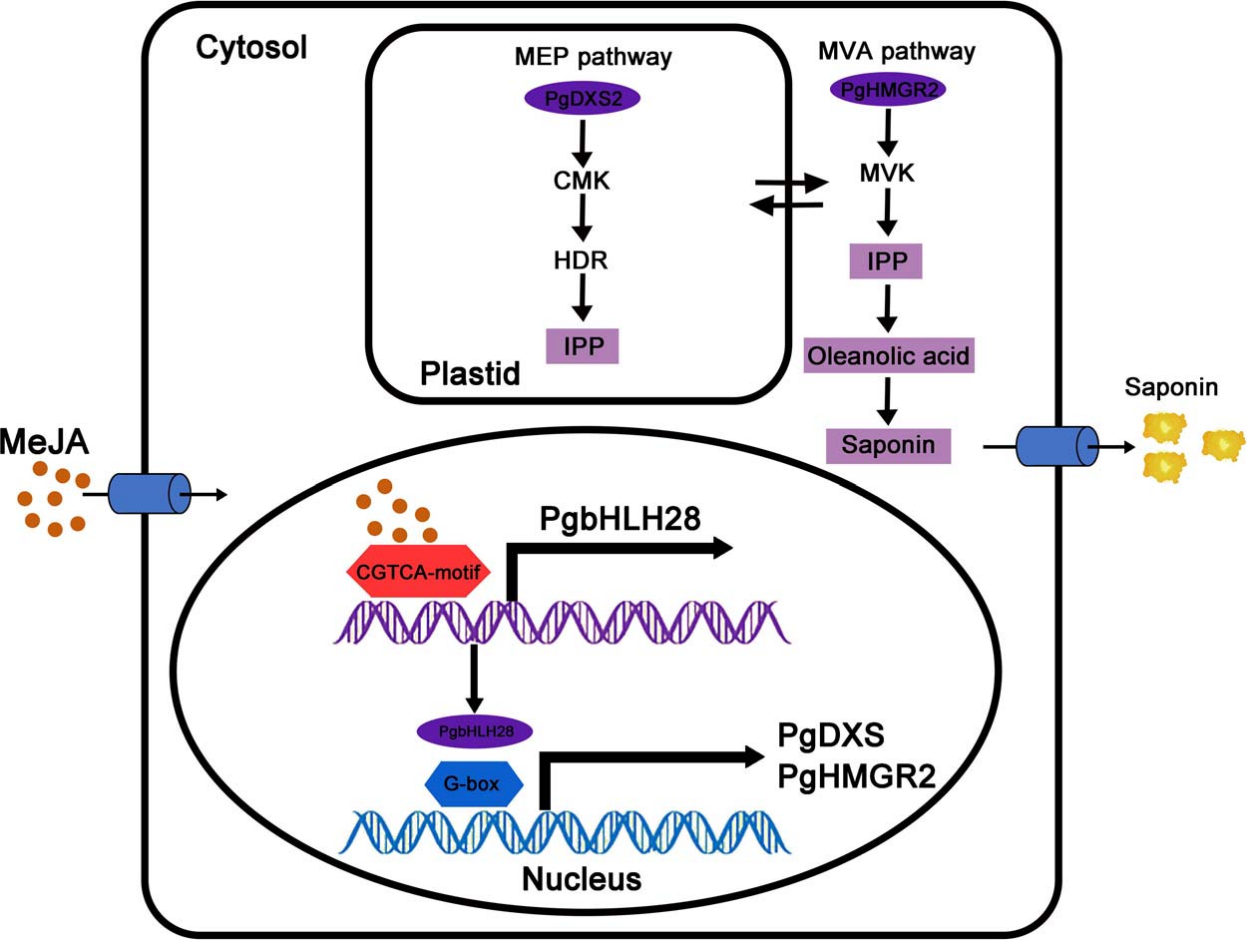
Working model of *PgbHLH28* in the regulation of saponin biosynthesis in *P. grandiflorus. PgbHLH28* specifically binds with the promoters of the key structural genes involved in saponin biosynthesis (including *PgDXS2* and *PgHMGR2*) and activates their transcription, resulting in enhanced saponin accumulation in *P. grandiflorus*. Furthermore, intermediate products produced by both the MVA and MEP pathways can be utilized in both pathways via transmembrane transport.

*PgDXS2* and *PgHMGR2* serve as critical rate-limiting enzymes in the MEP and MVA pathways for saponin biosynthesis [23]. *PgbHLH28* gene overexpression was found to promote saponin accumulation. The dual-luciferase reporter assays, GUS staining, and Y1H experiments proved that the *PgbHLH28* gene could bind to the promoters of *PgDXS2* and *PgHMGR2* genes to regulate their expression. To validate the functions of key rate-limiting enzymes PgDXS2 and PgHMGR2, overexpression and RNA interference vectors for two rate-limiting enzymes were constructed as described in above methods in this article. The vectors were then transformed into the *P. grandiflorus via A. tumefaciens* K599, yielding positive transgenic hairy roots (Figs. 7A–C and 8A–C). Transgenic hairy roots were assayed for total saponin content, intermediate product β-amyrin, and oleanolic acid content. The results demonstrated that these components were significantly higher in the transgenic hairy roots than in WT. In contrast, in silenced hairy roots, the content of these three components was significantly lower than that in WT (Figs. 8F and 9F). Gene expression data revealed that saponin biosynthesis pathway genes were upregulated in overexpressed hairy roots and suppressed in silenced hairy roots (Figs. 7D–E and 8D–E). These results indicated that both *PgDXS2* and *PgHMGR2* could enhance the saponin accumulation in *P. grandiflorus.*

## 4. Discussion

Specific concentrations of exogenous MeJA enhanced secondary metabolite content in medicinal plants. In *Leucas aspera* and *Panax quinquefolium*, exogenous MeJA increased oleic acid, triterpenoid, and ginsenoside content [24, 25]. However, different concentrations of MeJA exhibit varying effects on the content of the secondary metabolites [26]. Four concentration gradients were examined to investigate the influence of MeJA on saponin accumulation and identify the optimal inducing concentrations. The four different concentrations increased saponin content (Fig. 1B). When the concentration of exogenous MeJA was 100 μmol/l, the induction effect was optimal, and the saponin content was highest (Fig. 1B), similar to previous studies in *P. ginseng* [27, 28]. Therefore, this concentration can be applied in practical production to enhance saponin content in *P. grandiflorus*. Soluble sugars and starch, acting as substrates in glycolysis, directly impact the biosynthesis and accumulation of saponins via MVA and MEP pathways [1]. After MeJA treatment, the soluble sugar and starch content decreased compared to the control (Fig. 1C), while saponin content showed an increasing trend, suggesting that MeJA accelerated the conversion of soluble sugar and starch to saponins. Due to the differences in MeJA-induced accumulation patterns of various metabolites [26, 29], the content of various saponin monomers and intermediate biosynthesis products under 100 μmol/l treatment was measured. A significant increase was observed in β-amyrin and oleanolic acid content (Fig. 1C). The triterpenoid saponins in *P. grandiflorus* belong to the oleanane type, with oleanolic acid as the main sapogenin [30]. Therefore, total saponin content was directly promoted due to increased oleanolic acid content (Fig. 1C). Various saponin monomers are formed via hydroxylation, oxidation, and glycosylation of β-amyrin and oleanolic acid by the actions of *CYP450* and *GTs* [3]. Measurements of the content of the three saponin monomers identified that platycodin D and platycoside E exhibited an increasing trend while platycodin D3 was suppressed (Fig. 1C). MeJA may reduce platycodin D3 content by inhibiting the expression of genes that modify of platycodin D3, including *PgCYP450* and *PgUGT* genes (Supplementary Table S4). MeJA not only affected secondary metabolite content in plants but also had inhibitory effects on root growth [31, 32]. The MeJA-treated roots of *P. grandiflorus* were browned, and the degree of browning deepened with increasing MeJA concentration, demonstrating root growth inhibition (Fig. 1A).

Recent advances in high-throughput sequencing have made big data analysis a key approach for exploring genes involved in triterpenoid saponin biosynthesis. Preliminary transcriptomic analyses have been conducted on triterpenoid saponin biosynthetic pathways across different plant species, including *Glycyrrhiza uralensis* [33], *P. ginseng* [34], and *Clinopodium gracile* [35]. These studies have enhanced the gene library resources for triterpenoid saponin biosynthesis. KEGG enrichment and WGCNA analysis of MeJA-treated *P. grandiflorus* root transcriptomic data (Figs. 2A–C, 3A–C) identified the alpha-linolenic acid metabolism pathway, which was involved in the MeJA biosynthesis pathway [36], the glycolysis/gluconeogenesis, and starch and sucrose metabolism, which provide the key substrates pyruvic acid, glyceraldehyde 3-phosphate, and acetyl-coenzyme A for the MVA and MEP pathways [1]. Significant enrichment was also observed in the triterpene saponin backbone biosynthesis and the ABC transporters pathways, which were involved in saponin transportation and biosynthesis [37]. These data suggested that MeJA induced the expression of genes related to the glycolysis/gluconeogenesis, starch and sucrose metabolism, and triterpene saponin backbone synthesis pathway to promote the biosynthesis of triterpene saponins that finally activate the expression of genes related to ABC transporters pathway involved in transporting saponins from the cytoplasm to the extracellular compartment to increase saponin content in the roots.

The bHLH TFs play significant roles in the biosynthesis of terpenoid metabolites triggered by MeJA signaling, including monoterpenes, sesquiterpenes, diterpenes, and triterpenes [38, 39]. RNA-seq analysis revealed that the *PgbHLH28* gene exhibited upregulation in response to MeJA induction and held a central position in the regulatory network (Fig. 6A). In *Medicago truncatula*, TSAR1 (triterpene saponin biosynthesis activating regulator 1) and TSAR2 activation of *bAS*, *CYP93E2*, and *CYP72A61v2* genes increased hemolytic saponin content [13]. TSARL1 and TSARL2 also enhanced the accumulation of saponin content by activating *HMGR1* and *MKB1* genes [40]. Furthermore, bHLH TFs that regulate triterpenoid biosynthesis have been identified in *Cucumis sativus* L., *P. ginseng*, and *B. platyphylla* [17]. These data suggest that bHLH TFs participate in regulating saponin accumulation. To confirm *PgbHLH28* function, overexpression and RNA interference experiments were performed. The results demonstrated that *PgbHLH28* overexpression increased saponin accumulation and the expression of structural genes involved in saponin biosynthesis, while RNA silencing had the reverse effect (Fig. 5F–H). In addition to promoting saponin accumulation, some bHLH TFs also inhibit the accumulation of related metabolites. SmbHLH60 suppressed the transcription of *SmTAT1* and *SmDFR*, thereby inhibiting the biosynthesis of the phenolic acids and anthocyanins [41]. In *Salvia miltiorrhiza*, *SmbHLH92*-RNAi transgenic hairy roots showed increased salvianolic acid and tanshinone content, indicating that *SmbHLH92* acts as a negative regulator [42]. We also identified a negatively regulated transcription factor, *PgbHLH36*, in the regulatory network that may inhibit saponin synthesis by negatively regulating the saponin biosynthesis structural gene *PgHMGR1* (Supplementary Table S7). These results emphasize the complex nature of the bHLH gene family in regulating metabolic processes and the necessity of identifying the underlying transcriptional regulatory mechanisms of saponin biosynthesis.

Terpenoids represent the largest group of secondary metabolites in plants [43]. In higher plants, the basic five-carbon units of all terpenoids, IPP, and dimethylallyl pyrophosphate (DMAPP), are synthesized via two independent pathways in different cellular compartments. Monoterpenes, diterpenes, and tetraterpenes are products from the MEP pathway, which is located in the cytoplasm. Sesquiterpenes, sterols, and triterpenes are derivatives from the mevalonate (MVA) pathway located in the plastids [44]. Despite their separation in different organelles, evidence shows intermediates from these pathways can cross the plastid membrane, indicating mutual utilization [45]. Isotopic labeling of intermediates in *Antirrhinum majus* L. and *Solidago canadensis* L., sesterterpenes (primarily synthesized by the MVA pathway), rely on intermediates produced by the MEP pathway, which is primarily responsible for generating monoterpenes. This demonstrates that the intermediates between the two pathways can be exchanged and utilized through the plastid membrane [45–47]. Transcriptome analysis of MeJA-treated roots revealed that genes involved in the MEP and MVA pathways were induced and upregulated to varying degrees (Fig. 4A–B). Furthermore, the analysis revealed that *PgDXS2* and *PgHMGR2*, encoding enzymes in the MEP and MVA pathways, respectively, were the core structural genes in the regulatory network, suggesting that both pathways may be involved in saponin biosynthesis (Fig. 6A). Overexpression of *PgDXS2* and *PgHMGR2* resulted in elevated content of oleanolic acid, β-amyrin, and total saponins, accompanied by upregulation of saponin biosynthesis pathway genes (Figs. 7 and 8). In contrast, inhibition of these genes suppressed intermediate metabolite levels, as well as the expression levels of pathway genes. These findings suggested that the intermediates of the two pathways may be mutually utilized through the plastid membrane, synthesizing downstream terpenoids in *P. grandiflorus*.

## 5. Conclusion

This study aimed to elucidate the underlying molecular mechanisms of the role of MeJA in promoting saponin biosynthesis in *P. grandiflorus*. RNA-seq data analysis demonstrated that *PgbHLH28* acts as a positive regulator of saponin biosynthesis. Y1H, dual-luciferase, and transgenic experiments showed that *PgbHLH28* promoted saponin accumulation by activating the expression of *PgHMGR2* and *PgDXS2*. Moreover, overexpression and silencing of *PgDXS2* and *PgHMGR2* demonstrated that both MVA and MEP pathways contribute to saponin biosynthesis, indicating the pathways’ intermediates are mutually utilized through the plasma membrane (Fig. 9). These research results have increased the understanding of the saponin biosynthesis mechanism induced by MeJA. These findings, therefore, support future research on saponin biosynthesis and molecular breeding strategies.

## 2. Materials and methods

### 2.1. Plant material and measurements

*Platycodon grandiflorus* plants used in this study were cultivated in Harbin, Heilongjiang Province, China. The sterile seedlings cultivated in the laboratory for 90 days were treated separately with MeJA concentrations: 0, 10, 50, 100, or 200 μM. The samples were collected at 0, 12, 24, and 48 hours, immediately frozen in liquid nitrogen, and stored at −80°C for further transcriptome and metabolite analyses. Total saponin content was measured using the vanillin–perchloric acid colorimetry assay [48]. Starch and soluble sugars were determined using the anthrone–sulfuric acid colorimetric assay [49, 50]. To ensure validity, three biological replicates per sample per experiment were performed. Each replicate contained 10 plants of uniform size.

### 2.2. Oleanolic acid, β-amyrin, and sapogenin monomers content analysis in *P. grandiflorus*

Oleanolic acid, β-amyrin and three saponin monomers (platycoside E, platycodin D3, and platycodin D) standards were purchased from Chengdu Desite Biotechnology Co., Ltd. (Chengdu, China). Metabolites were extracted from 0.5 g of *P. grandiflorus* root treated with MeJA concentrations of 0, 10, 50, 100, or 200 μM by adding 1 ml of methanol, sonicating for 30 min, centrifuging at 12 000 rpm for 10 min at 4°C, and filtering through a 0.22-μm syringe membrane for High-Performance Liquid Chromatography (HPLC) analysis. The HPLC detection was performed on the Agilent Series1260 system (Agilent Technologies Inc., USA), with Techmate C18 column (250 × 4.6 mm, 5 μm). The mobile phase for oleanolic acid and β-amyrin was methanol (A)-water (B) (90:10 v/v), with detection wavelengths of 203 and 215 nm, respectively. The mobile phase for saponin monomers was acetonitrile (A)-water (B) (27:73 v/v), with a detection wavelength of 215 nm. The sample injection volume was 10 μl, the flow rate was 1 ml/min, column temperature was 30°C [51]. Each sample was performed with three biological replicates.

### 2.3. RNA sequencing (RNA-seq) and data analysis

Total RNA from *P. grandiflorus* roots treated with 100 μM MeJA for 0, 12, 24, or 48 h was extracted using the RNeasy Plant Mini Kit (Qiagen, Venlo, Netherlands). The concentration and quality of the extracted RNA were assessed using the GXII Touch HT Nucleic Acid Analyzer (PerkinElmer, USA). After passing the quality control, the RNA library was constructed for each sample using the KAPA™ Single-Stranded RNA Library Preparation Kit (Illumina, USA). The libraries were sequenced on the Illumina HiSeq-2000 platform (Illumina, USA). The paired-end reads were mapped to the *P. grandiflorus* genome (https://ngdc.cncb.ac.cn/gwh) using HISAT2 [52]. Then, featureCounts was used to calculate transcripts per million (TPM) [53]. To ensure experimental accuracy, three biological replicates were used for RNA-seq and subsequent bioinformatic analyses. Each biological replicate included 10 individual plants of uniform size. The clean reads of 12 samples from this study were submitted to NCBI (accession number: PRJNA1032698).

### 2.4. Construction of saponin biosynthesis regulatory network under MeJA treatment

Differentially expressed genes (DEGs) (TPM > 1 and coefficient of variation (CV) > 0.1) were used to generate a regulatory network for saponin biosynthesis under MeJA treatment using the weighted correlation network analysis (WGCNA) package. The parameters were determined based on the previously described method [54]. The initial modules were merged using eigengenes to identify the key modules regulating saponin biosynthesis. This package was then used to calculate eigengene values for each module, to establish the relationship between metabolites and the modules associated with saponin biosynthesis. The Muffuz package [55] was used to partition the selected module sets into different clusters based on expression similarity patterns to obtain the core genes responsible for regulating saponin biosynthesis under MeJA treatment.

### 2.5. qRT-PCR

Gene expression analysis via quantitative reverse transcription polymerase chain reaction (qRT-PCR) was performed according to the established protocols, including RNA extraction, reverse transcription, PCR amplification, and data analysis [20]. Three biological replicates per sample were analyzed with the *18S RNA* gene as the internal control [1]. The gene-specific primers for qRT-PCR were presented in Supplementary Table S1.

### 2.6. Yeast one-hybrid assay

Yeast one-hybrid assay (Y1H) assay was performed using the pLacZi One-Hybrid system. The full-length *PgbHLH28* TF was inserted between *Kpn* I and *Xho* I sites in the pB42AD vector as the bait. The promoters of *PgUGT85A4*, *PgDXS2*, *PgHMGR2*, *PgHMGR1*, and *PgUGT90A1* genes were ligated between *Kpn* I and *Xho* I sites in the pLacZi vector as the prey. The bait and prey vectors were then co-transformed into yeast strain EGY48. The pB42AD-*PgbHLH28* and an empty vector pLacZi were used as controls. The SD/−Trp/-Ura medium was used for the growth of transformants. After 3 days, yeast-exhibiting positive clones were transferred to SD/−Trp/-Ura yeast media containing X-Gal (5-bromo-4-chloro-3-indolyl-β-D-galactopyranoside) and cultured in the dark at 30°C for 2–3 days. The color changes of the recombinant yeast on the agar plates were examined to identify an interaction between the *PgbHLH28* and the downstream functional genes [56].

### 2.7. Dual-luciferase assay

The *PgbHLH28* coding sequence was inserted between *Bam*H I and *Hin*d III sites in the pGreenII62-SK vector as the effector. *PgDXS2* and *PgHMGR2* promoter sequences were inserted between *Bam*H I and *Hin*d III sites in the pGreen 0800-LUC vector as the double-reporter. The constructed reporter and effector plasmids were transformed into *Agrobacterium tumefaciens* strain GV3101 (psoup-p19). Cell suspensions with effector and reporter vectors were then transiently injected into 4-week-old tobacco leaves [57, 58]. The pGreen 0800-ProPgDXS2-LUC and pGreen 0800-ProPgHMGR2-LUC vectors were used as the control. After 2–3 days of cultivation, the results of the dual-luciferase experiments were photographed using an automated chemiluminescence image analysis system (Tanon 5200, Shanghai, China), and the LUC/REN activity was measured using a Luciferase Reporter Gene Assay Kit (Yeasen Biotechnology, Shanghai, China) [59]. Three biological replicates were performed for the dual-luciferase assay. Each biological replicate included five different tobacco leaves.

### 2.8. *PgbHLH28* overexpression in *P. grandiflorus*

The full-length *PgbHLH28* gene, lacking a termination codon, was inserted between *Bam*H I and *Kpn* I sites in the pCAMBIA1300-sGFP overexpression vector. The correctly sequenced pCAMBIA1300-*PgbHLH28*-sGFP vector was then used in the *A. tumefaciens* GV3101 transformation. *Platycodon grandiflorus* leaf explants were precultured on differentiation medium (MS medium containing 1 mg/l 6-BA, 0.1 mg/l NAA, 30 g/l sucrose, and 5 g/l agar, at pH 5.8) for 7 days and then transferred into *A. tumefaciens* GV3101 cell suspension containing the pCAMBIA1300-*PgbHLH28*-sGFP construct (OD = 0.6). After 20 min of infection at 28°C, the excess bacterial solution was removed using filter paper and co-cultured for 4 days under dark conditions. The explants were then transferred to a shoot-inducing medium (MS medium containing 1 mg/l 6-BA, 0.1 mg/l NAA, 30 g/l sucrose, 5 g/l agar, 30 mg/l hygromycin B, and 400 mg/l cefotaxime sodium). Upon shoot formation on the explant cultures, the shoots were transferred to selective rooting medium (1/2 MS medium containing 0.5 mg/l NAA, 30 g/l sucrose, 5 g/l agar, 30 mg/l hygromycin B, and 400 mg/l cefotaxime sodium) to induce root formation. Each separate shoot represented an independent line. After ~30 days, the rooted plants exhibiting hygromycin resistance were transferred to pots containing a 1:1 volume ratio of peat and vermiculite for growth under a 16/8 h light/dark photoperiod. Positive transgenic plants were identified according to the following three steps. Firstly, GFP fluorescence was detected in hairy root using an automated chemiluminescence image system (Tanon 5200, Shanghai, China) to confirm their transgenic status. Secondly, the RNA was extracted from positive transgenic hairy roots and amplified using *GFP* and *18S RNA* primers. Lastly, qRT-PCR was used to quantify transcript levels of *PgbHLH28* and related pathway genes. Primers used in this study are presented in Supplementary Table S7. HPLC and vanillin-perchloric acid colorimetry assay were used to measure saponin content as described in this article.

### 2.9. Functional verification of *PgbHLH28*, *PgDXS2*, and *PgHMGR2* genes using hairy root

The full-length *PgbHLH28, PgDXS2* and *PgHMGR2* genes, without termination codons, were inserted between *Bam*H I and *Kpn* I sites in the pCAMBIA1300-sGFP overexpression vector. The 300-bp specific forward and reverse fragments of *PgbHLH28*, *PgDXS2*, and *PgHMGR2* genes were inserted into the pFGC1008 vector between *Asc* I and *Bam*H I, as well as *Spe* I and *Swa* I sites to construct RNA interference vectors, respectively. The correctly sequenced overexpression and RNA interference vectors were transformed into *A. tumefaciens* strain K599, then cultivated on LB solid medium (containing 50 mg/l kanamycin) for 36–48 h. Selected single colonies were cultured in 5 ml of LB liquid medium, followed by overnight incubation in 200 ml of LB medium containing 50 mg/l kanamycin and 200 μmol/l AS. When the OD600 of the culture reached ~0.8, it was centrifuged at 4000 rpm for 10 min. The pellet was suspended in an infiltration buffer (containing 200 μmol/l AS, 10 mmol/l MES, and 10 mmol/l MgCl_2_) and then allowed to activate by incubating for 2 h at room temperature in the dark. This infiltration buffer was then used for infiltration. Thirty-day-old seedlings without roots were submerged in infiltration buffer twice and subjected to vacuum infiltration at −0.05 MPa, each for 90 s. The infected plants were washed with distilled water before transplanting into a 1:1 (v/v) mixture of peat and vermiculite for hairy root induction [60, 61]. Positive plant identification was conducted 60 days later. Transgenic hairy roots were detected using GFP green fluorescence detection and PCR. Transcript levels of *PgbHLH28*, *PgDXS2*, *PgHMGR2*, and related pathway genes in positive hairy roots were quantified using qRT-PCR. HPLC was used to measure the saponin content. Primers used in this study were presented in Supplementary Table S7.

### 2.10. GUS activity staining

The full-length *PgbHLH28* gene lacking termination codons was inserted between *Bam*H I and *Kpn* I sites in the pCAMBIA1300-sGFP overexpression vector. The 2000-bp sequences of both *PgDXS2* and *PgHMGR2* promoters were inserted between *Bam*H I and *Hin*d III sites in the pBI121-GUS vector. The recombinant vector pCAMBIA1300-*PgbHLH28*-sGFP, proPgDXS2-GUS, and proPgHMGR2-GUS were transformed into GV3101. The pCAMBIA1300-*PgbHLH28*-sGFP and proPgDXS2-GUS, proPgHMGR2-GUS were respectively co-expressed in the 0.5-cm-thick root segments submerged in the cell suspension (containing 200 μmol/l AS, 10 mmol/l MES, and 10 mmol/l MgCl_2_) for 20 min. The excess bacterial solution was removed with filter paper, and the treated root segments were transferred onto plates for incubation in the dark for 3 days. For histochemical staining, the root segments were immersed in GUS staining buffer (2 mM ferricyanide, 0.1% Triton X-100, 10 mM EDTA, 2 mM ferricyanide, phosphate buffer, and 2 mM X-Gluc, at pH 7.2) at 37°C overnight under dark conditions. Finally, the color changes and GUS activity were examined to determine interactions between the *PgbHLH28* and the downstream structural genes *PgDXS2* and *PgHMGR2*.

### 2.11. Statistical analysis

All experiments were conducted with three biological replicates. Results are reported as mean ± standard deviation (SD). Statistical significance was indicated by asterisks, as determined by Student’s *t*-test or one-way analysis of variance (*, *P* < .05).

## Supplementary Material

Web_Material_uhae058

## Data Availability

The data presented in this study are available in the article or supplementary material. The raw RNA-seq data generated in this study are available in the NCBI-SRA database (PRJNA1032698). The sequences of *PgbHLH28*, *PgDXS2* and *PgHMGR2* are available in the NGDC database (C_AA057966.1- C_AA057968.1).

## References

[1] Su X , Liu Y, Han L. et al. A candidate gene identified in converting platycoside E to platycodin D from *Platycodon grandiflorus* by transcriptome and main metabolites analysis. Sci Rep. 2021;11:9810 33963244 10.1038/s41598-021-89294-1 PMC8105318

[2] Wu J , Yang G, Zhu W. et al. Anti-atherosclerotic activity of Platycodin D derived from roots of *Platycodon grandiflorum* in human endothelial cells. Biol Pharm Bull. 2012;35:1216–21 22863916 10.1248/bpb.b-y110129

[3] Kwon J , Lee H, Kim N. et al. Effect of processing method on platycodin D content in *Platycodon grandiflorum* roots. Arch Pharm Res. 2017;40:1087–93 28852980 10.1007/s12272-017-0946-6

[4] Sharma R , Palanisamy A, Dhama K. et al. Exploring the possible use of saponin adjuvants in COVID-19 vaccine. Hum Vaccin Immunother. 2020;16:2944–53 33295829 10.1080/21645515.2020.1833579 PMC7738204

[5] Ghaemi A , Roshani Asl P, Zargaran H. et al. Recombinant COVID-19 vaccine based on recombinant RBD/nucleoprotein and saponin adjuvant induces long-lasting neutralizing antibodies and cellular immunity. Front Immunol. 2022;13:974364 36159845 10.3389/fimmu.2022.974364 PMC9494508

[6] Li Y , Tan C, Li Z. et al. The genome of Dioscorea zingiberensis sheds light on the biosynthesis, origin and evolution of the medicinally important diosgenin saponins. Horticulture Research. 2022;9:uhac165 36204203 10.1093/hr/uhac165 PMC9531337

[7] Zhao CL , Cui XM, Chen YP. et al. Key enzymes of triterpenoid saponin biosynthesis and the induction of their activities and gene expressions in plants. Nat Prod Commun. 2010;5:1147–58 20734961

[8] Meng F , Chu T, Feng P. et al. Genome assembly of Polygala tenuifolia provides insights into its karyotype evolution and triterpenoid saponin biosynthesis. Horticulture Research. 2023;10:uhad139 37671073 10.1093/hr/uhad139 PMC10476160

[9] Moses T , Pollier J, Almagro L. et al. Combinatorial biosynthesis of sapogenins and saponins in *Saccharomyces cerevisiae* using a C-16α hydroxylase from *Bupleurum falcatum*. Proc Natl Acad Sci USA. 2014;111:1634–9 24434554 10.1073/pnas.1323369111 PMC3910630

[10] Kim Y-J , Lee OR, Oh JY. et al. Functional analysis of 3-hydroxy-3-methylglutaryl coenzyme a reductase encoding genes in triterpene saponin-producing ginseng. Plant Physiol. 2014;165:373–87 24569845 10.1104/pp.113.222596 PMC4012596

[11] Xue Z , Duan L, Liu D. et al. Divergent evolution of oxidosqualene cyclases in plants. New Phytol. 2012;193:1022–38 22150097 10.1111/j.1469-8137.2011.03997.x

[12] Li Y , Lin Y, Jia B. et al. Transcriptome analysis reveals molecular mechanisms underlying methyl jasmonate-mediated biosynthesis of protopanaxadiol-type saponins in *Panax notoginseng* leaves. J Plant Biol. 2022;65:29–41

[13] Ribeiro B , Lacchini E, Bicalho KU. et al. A seed-specific regulator of triterpene saponin biosynthesis in *Medicago truncatula*. Plant Cell. 2020;32:2020–42 32303662 10.1105/tpc.19.00609 PMC7268793

[14] Singh AK , Kumar SR, Dwivedi V. et al. A WRKY transcription factor from *Withania somnifera* regulates triterpenoid withanolide accumulation and biotic stress tolerance through modulation of phytosterol and defense pathways. New Phytol. 2017;215:1115–31 28649699 10.1111/nph.14663

[15] Hong G-J , Xue X-Y, Mao Y-B. et al. *Arabidopsis* MYC2 interacts with DELLA proteins in regulating sesquiterpene synthase gene expression. Plant Cell. 2012;24:2635–48 22669881 10.1105/tpc.112.098749 PMC3406894

[16] Yamamura C , Mizutani E, Okada K. et al. Diterpenoid phytoalexin factor, a bHLH transcription factor, plays a central role in the biosynthesis of diterpenoid phytoalexins in rice. Plant J. 2015;84:1100–13 26506081 10.1111/tpj.13065

[17] Shang Y , Ma Y, Zhou Y. et al. Plant science. Biosynthesis, regulation, and domestication of bitterness in cucumber. Science. 2014;346:1084–8 25430763 10.1126/science.1259215

[18] Zhang X , Ge F, Deng B. et al. Molecular cloning and characterization of PnbHLH1 transcription factor in *Panax notoginseng*. Molecules. 2017;22:1268 28758911 10.3390/molecules22081268 PMC6152055

[19] Chu Y , Xiao S, Su H. et al. Genome-wide characterization and analysis of bHLH transcription factors in *Panax ginseng*. Acta Pharm Sin B. 2018;8:666–77 30109190 10.1016/j.apsb.2018.04.004 PMC6089850

[20] Yin J , Li X, Zhan Y. et al. Cloning and expression of *BpMYC4* and *BpbHLH9* genes and the role of *BpbHLH9* in triterpenoid synthesis in birch. BMC Plant Biol. 2017;17:214 29162040 10.1186/s12870-017-1150-z PMC5698961

[21] Li N , Bo C, Zhang Y. et al. PHYTOCHROME INTERACTING FACTORS PIF4 and PIF5 promote heat stress induced leaf senescence in Arabidopsis. J Exp Bot. 2021;72:4577–89 33830198 10.1093/jxb/erab158 PMC8446286

[22] Cao X , Jiang D, Wang H. et al. Identification of UGT85A glycosyltransferases associated with volatile conjugation in grapevine (*Vitis vinifera × Vitis labrusca*). Horticultural Plant Journal. 2023;9:1095–107

[23] Leng X , Cong J, Cheng L. et al. Identification of key gene networks controlling monoterpene biosynthesis during grape ripening by integrating transcriptome and metabolite profiling. Horticultural Plant Journal. 2023;9:931–46

[24] Kochan E , Balcerczak E, Lipert A. et al. Methyl jasmonate as a control factor of the *synthase squalene* gene promoter and ginsenoside production in American ginseng hairy root cultured in shake flasks and a nutrient sprinkle bioreacto. Ind Crop Prod. 2018;115:182–93

[25] Vijendra PD , Jayanna SG, Kumar V. et al. Product enhancement of triterpenoid saponins in cell suspension cultures of *Leucas aspera* Spreng. Ind Crop Prod. 2020;156:112857

[26] Ebrahim B , Leyla H, Babak AM. et al. The effect of different concentrations of methyl jasmonate on the activity of antioxidant enzymes and total protein in basil. Journal of Crops lmprovement. 2016;18:103–15

[27] Han JY , Kim HJ, Kwon YS. et al. The Cyt P450 enzyme CYP716A47 catalyzes the formation of protopanaxadiol from dammarenediol-II during ginsenoside biosynthesis in *Panax ginseng*. PLANT CELL PHYSIOL. 2011;52:2062–73 22039120 10.1093/pcp/pcr150

[28] Han J-Y , In J-G, Kwon Y-S. et al. Regulation of ginsenoside and phytosterol biosynthesis by RNA interferences of squalene epoxidase gene in *Panax ginseng*. Phytochemistry. 2010;71:36–46 19857882 10.1016/j.phytochem.2009.09.031

[29] Fard FR , Omidbaigi R, Sharifi M. et al. Effect of methyl jasmonate on essential oil content and composition of *Agastache foeniculum*. Academic Journals. 2012;6:5701–5

[30] Kim J , Kang S-H, Park S-G. et al. Whole-genome, transcriptome, and methylome analyses provide insights into the evolution of platycoside biosynthesis in *Platycodon grandiflorus*, a medicinal plant. Horticulture Research. 2020;7:112 32637140 10.1038/s41438-020-0329-x PMC7327020

[31] Sohn S-I , Pandian S, Rakkammal K. et al. Jasmonates in plant growth and development and elicitation of secondary metabolites: an updated overview. Front Plant Sci. 2022;13:942789 36035665 10.3389/fpls.2022.942789 PMC9407636

[32] Yan J , Zhang C, Gu M. et al. The *Arabidopsis* CORONATINE INSENSITIVE1 protein is a jasmonate receptor. Plant Cell. 2009;21:2220–36 19717617 10.1105/tpc.109.065730 PMC2751961

[33] Li Y , Luo H-M, Sun C. et al. EST analysis reveals putative genes involved in glycyrrhizin biosynthesis. BMC Genomics. 2010;11:268 20423525 10.1186/1471-2164-11-268 PMC2886062

[34] Chen S , Luo H, Li Y. et al. 454 EST analysis detects genes putatively involved in ginsenoside biosynthesis in *Panax ginseng*. Plant Cell Rep. 2011;30:1593–601 21484331 10.1007/s00299-011-1070-6

[35] Shan C , Wang C, Zhang S. et al. Transcriptome analysis of *Clinopodium gracile* (Benth.) Matsum and identification of genes related to triterpenoid saponin biosynthesis. BMC Genomics. 2020;21:49 31941462 10.1186/s12864-020-6454-y PMC6964110

[36] Huan C , Yang X, Wang L. et al. Methyl jasmonate treatment regulates α-linolenic acid metabolism and jasmonate acid signaling pathway to improve chilling tolerance in both stony hard and melting flesh peaches. Postharvest Biol Technol. 2022;190:111960–5214

[37] Fang Y , Xiao H. The transport of triterpenoids. Biotechnology Notes. 2021;2:11–7

[38] Goossens J , Mertens J, Goossens A. Role and functioning of bHLH transcription factors in jasmonate signalling. J Exp Bot. 2017;68:1333–47 27927998 10.1093/jxb/erw440

[39] Man J , Shi Y, Huang Y. et al. PnMYB4 negatively modulates saponin biosynthesis in Panax notoginseng through interplay with PnMYB1. Horticulture Research. 2023;10:10 10.1093/hr/uhad134 PMC10410195 37564268

[40] Tamura K , Yoshida K, Hiraoka Y. et al. The basic helix–loop–helix transcription factor GubHLH3 positively regulates soyasaponin biosynthetic genes in *Glycyrrhiza uralensis*. Plant Cell Physiol. 2018;59:783–96 10.1093/pcp/pcy046 29648666

[41] Liu S , Wang Y, Shi M. et al. SmbHLH60 and SmMYC2 antagonistically regulate phenolic acids and anthocyanins biosynthesis in *salvia miltiorrhiza*. J Adv Res. 2022;42:205–19 36513414 10.1016/j.jare.2022.02.005 PMC9788942

[42] Zhang J , Lv H, Liu W. et al. bHLH transcription factor *SmbHLH92* negatively regulates biosynthesis of phenolic acids and tanshinones in *salvia miltiorrhiza*. Chinese Herbal Medicines. 2020;12:237–46 36119017 10.1016/j.chmed.2020.04.001 PMC9476745

[43] Zhang Z , Wu Q-Y, Ge Y. et al. Hydroxylases involved in terpenoid biosynthesis: a review. Bioresour Bioprocess. 2023;10:39 38647640 10.1186/s40643-023-00656-1 PMC10992849

[44] Böttger A , Vothknecht U, Bolle C. et al. Terpenes and terpenoids. In: Böttger A, Vothknecht U, Bolle C, Wolf A, eds. Lessons on Caffeine, Cannabis & Co: Plant-Derived Drugs and their Interaction with Human Receptors. Springer International Publishing: Cham, 2018,153–70

[45] Laule O , Fürholz A, Chang H-S. et al. Crosstalk between cytosolic and plastidial pathways of isoprenoid biosynthesis in *Arabidopsis thaliana*. Proc Natl Acad Sci USA. 2003;100:6866–71 12748386 10.1073/pnas.1031755100 PMC164538

[46] Rodríguez-Concepción M . Early steps in isoprenoid biosynthesis: multilevel regulation of the supply of common precursors in plant cells. Phytochem Rev. 2006;5:1–15

[47] Bick JA , Lange BM. Metabolic cross talk between cytosolic and plastidial pathways of isoprenoid biosynthesis: unidirectional transport of intermediates across the chloroplast envelope membrane. Arch Biochem Biophys. 2003;415:146–54 12831836 10.1016/s0003-9861(03)00233-9

[48] Sun HD , Gao Y, An XL. et al. Optimization of the culture medium of adventitious root culture to produce the flavonoids and the triterpenoids of Actinidia arguta by using an orthogonal design process. Plant Cell Tissue Organ Cult. 2021;144:545–54

[49] Somani BL , Khanade J, Sinha R. A modified anthrone-sulfuric acid method for the determination of fructose in the presence of certain proteins. Anal Biochem. 1987;167:327–30 3442328 10.1016/0003-2697(87)90172-2

[50] Laurentin A , Edwards CA. A microtiter modification of the anthrone-sulfuric acid colorimetric assay for glucose-based carbohydrates. Anal Biochem. 2003;315:143–5 12672425 10.1016/s0003-2697(02)00704-2

[51] Yu H , Liu M, Yin M. et al. Transcriptome analysis identifies putative genes involved in triterpenoid biosynthesis in *Platycodon grandiflorus*. Planta. 2021;254:34 34291354 10.1007/s00425-021-03677-2

[52] Kim D , Langmead B, Salzberg SL. HISAT: a fast spliced aligner with low memory requirements. Nat Methods. 2015;12:357–60 25751142 10.1038/nmeth.3317 PMC4655817

[53] Liao Y , Smyth GK, Shi W. featureCounts: an efficient general purpose program for assigning sequence reads to genomic features. Bioinformatics. 2014;30:923–30 24227677 10.1093/bioinformatics/btt656

[54] Wang R , Shu P, Zhang C. et al. Integrative analyses of metabolome and genome-wide transcriptome reveal the regulatory network governing flavor formation in kiwifruit (*Actinidia chinensis*). New Phytol. 2022;233:373–89 34255862 10.1111/nph.17618

[55] Shen B , Yi X, Sun Y. et al. Proteomic and metabolomic characterization of COVID-19 patient sera. Cell. 2020;182:59–72.e15 32492406 10.1016/j.cell.2020.05.032 PMC7254001

[56] Xu S , Wu Z, Hou H. et al. The transcription factor CmLEC1 positively regulates the seed-setting rate in hybridization breeding of chrysanthemum. Horticulture Research. 2021;8:191 34376645 10.1038/s41438-021-00625-9 PMC8355372

[57] Xi H , He Y, Chen H. Functional characterization of *SmbHLH13* in anthocyanin biosynthesis and flowering in eggplant. Horticultural Plant Journal. 2021;7:73–80

[58] Dong B , Wang Q, Zhou D. et al. Abiotic stress treatment reveals expansin like a gene *OfEXLA1* improving salt and drought tolerance of *Osmanthus fragrans* by responding to abscisic acid. Horticultural Plant Journal. 2024;10:573–85

[59] Hao Y , Fan X, Shi Y. et al. Next-generation unnatural monosaccharides reveal that ESRRB O-GlcNAcylation regulates pluripotency of mouse embryonic stem cells. Nat Commun. 2019;10:4065 31492838 10.1038/s41467-019-11942-y PMC6731260

[60] Liu G , Yuan Y, Jiang H. et al. *Agrobacterium tumefaciens*-mediated transformation of modern rose (*Rosa hybrida*) using leaf-derived embryogenic callus. Horticultural Plant Journal. 2021;7:359–66

[61] Cao X , Xie H, Song M. et al. Cut-dip-budding delivery system enables genetic modifications in plants without tissue culture. Innovation (Camb). 2023;4:100345 36387605 10.1016/j.xinn.2022.100345 PMC9661722

