# Supplementary material for: RNA sequencing analysis reveals PgbHLH28 as the key regulator in response to methyl jasmonate-induced saponin accumulation in Platycodon grandiflorus
Source: Hortic Res. 2024 Feb 28;11(5):uhae058. doi: 10.1093/hr/uhae058 (PMC11070725; doi:10.1093/hr/uhae058)
Supplement: Web_Material_uhae058 [file web_material_uhae058.zip › Supplemental Material Figure.docx]

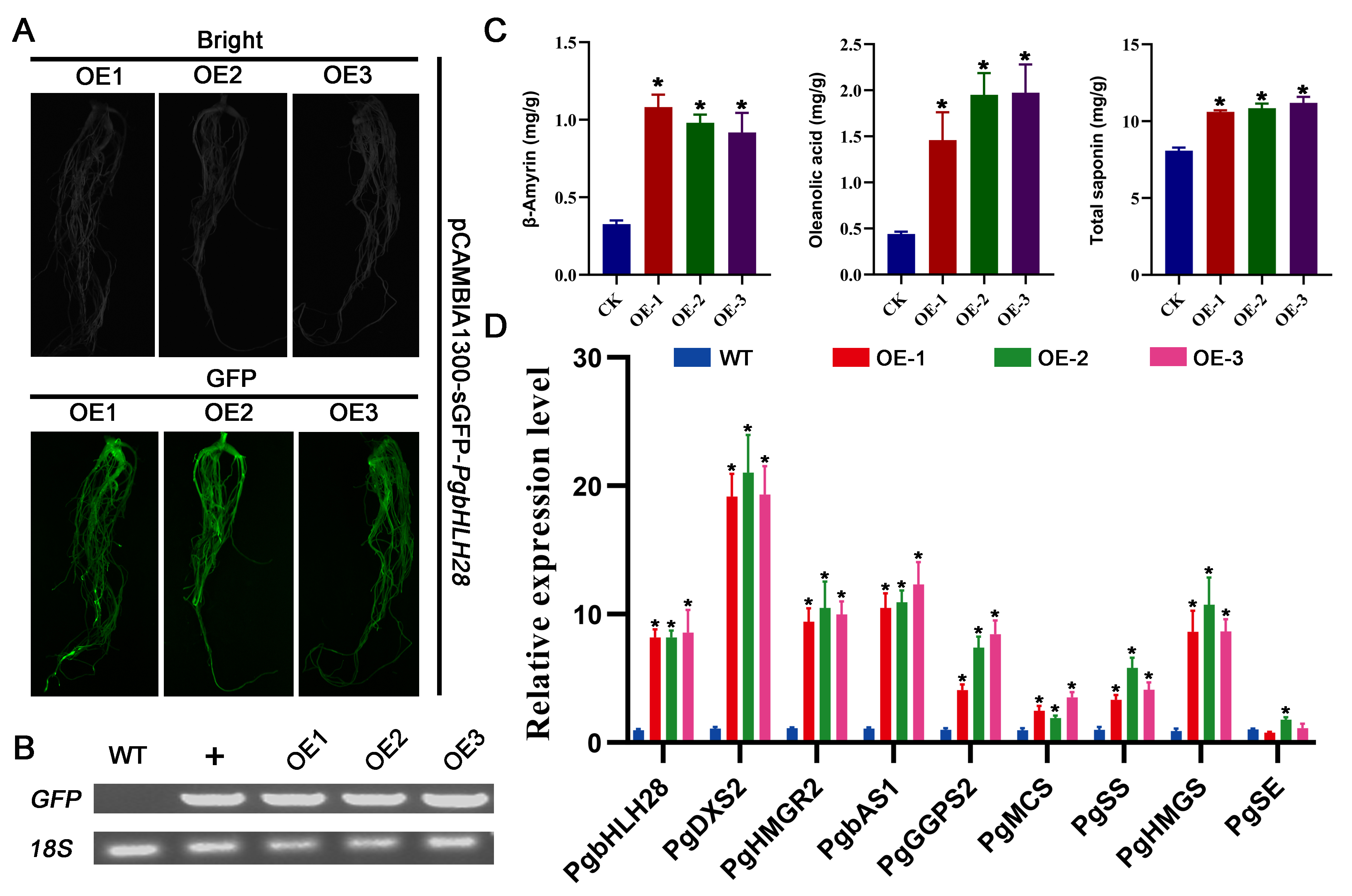


Figure S1. *PgbHLH28* modulates saponin accumulation in *P. grandiflorus*. (A) Hairy root generation through overexpression of *PgbHLH28* in *P. grandiflorus*. (B) RT-PCR validation of transgenic hairy root. (C) The content of total saponin, oleanolic acid, and β-amyrin in *PgbHLH28* overexpression lines. (D) Relative expression of genes involved in saponin biosynthesis in *PgbHLH28* overexpression lines. *PgbHLH28*-OE1, *PgbHLH28*-OE2, and *PgbHLH28*-OE3 are three independent *PgbHLH28* overexpression lines.
