# Supplementary figures and images for: RNA sequencing analysis reveals PgbHLH28 as the key regulator in response to methyl jasmonate-induced saponin accumulation in Platycodon grandiflorus
Source: Hortic Res. 2024 Feb 28;11(5):uhae058. doi: 10.1093/hr/uhae058 (PMC11070725; doi:10.1093/hr/uhae058)

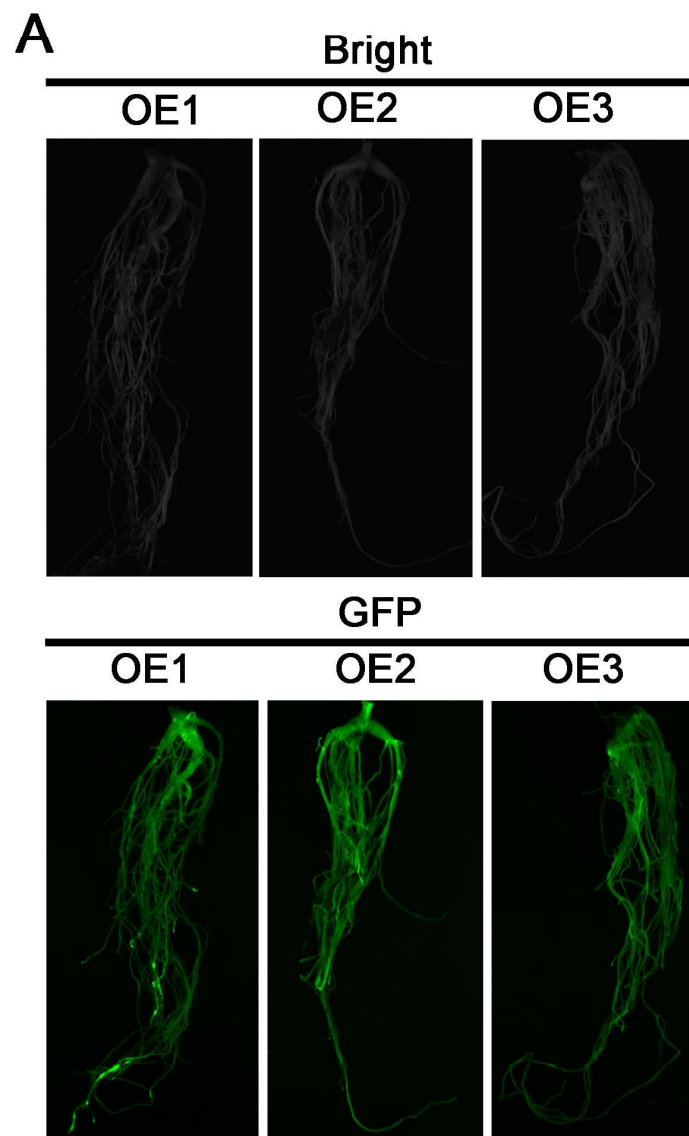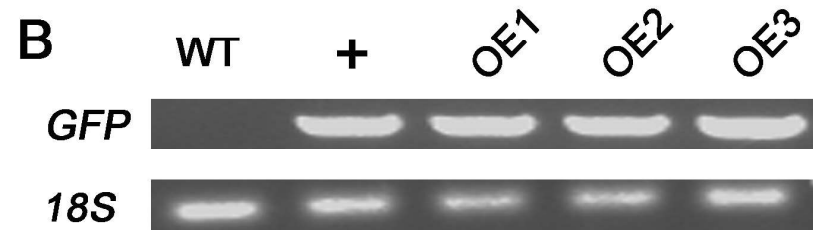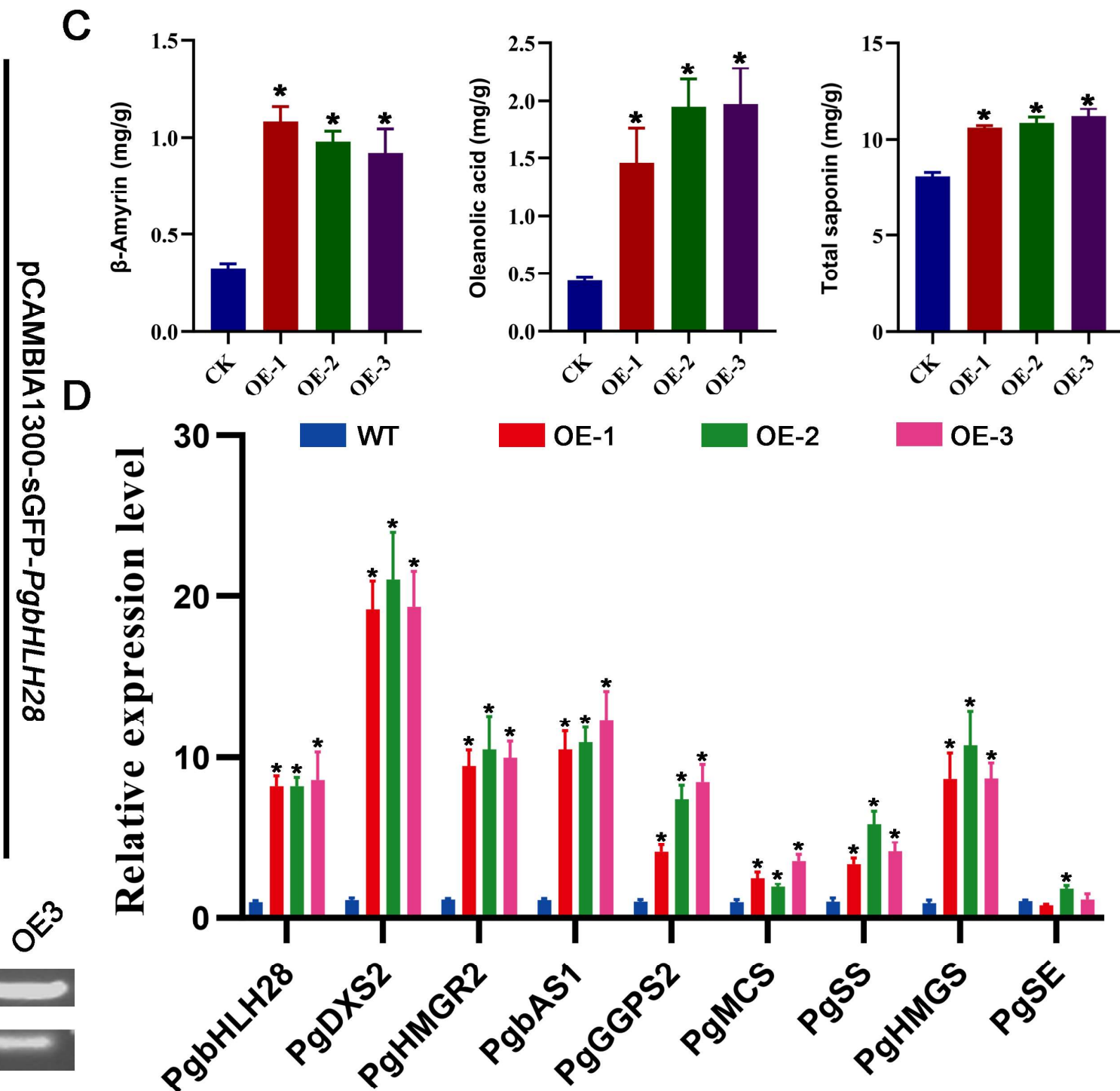

Supplement: Web_Material_uhae058 [file web_material_uhae058.zip › FigS1.pdf]
